# Normative Optical Coherence Tomography Angiography Metrics of Macular Vessel Density and Foveal Avascular Zone in Healthy Children

**DOI:** 10.3390/jcm14196911

**Published:** 2025-09-29

**Authors:** María Concepción Guirao-Navarro, Pablo Viñeta-Garcia, Javier Zarranz-Ventura, Jesús Barrio-Barrio

**Affiliations:** 1 Hospital Universitario Morales Meseguer, 30008 Murcia, Spain; mgnavarro@alumni.unav.es; 2 Clínica Guirao, 30004 Murcia, Spain; 3 Department of Ophthalmology, Clínica Universidad de Navarra, 31008 Pamplona, Spain; pvinetagarc@alumni.unav.es (P.V.-G.); jbarrio@unav.es (J.B.-B.); 4 Hospital Clínic de Barcelona, 08036 Barcelona, Spain; 5 Institut d’Investigacions Biomediques August Pi i Sunyer (IDIBAPS), 08036 Barcelona, Spain; 6 Facultat de Medicina, Universitat de Barcelona, 08036 Barcelona, Spain

**Keywords:** OCTA, normative, database, pediatric, children, vessel density, perfusion density, foveal avascular zone, metrics, angiolytics

## Abstract

**Background**: Optical coherence tomography angiography (OCTA) enables non-invasive, high-resolution visualization of the retinal microvasculature and is increasingly utilized in pediatric ophthalmology. However, its clinical application in children is limited by the absence of age-specific normative data. **Methods**: In this cross-sectional study, macular vessel density (VD) and foveal avascular zone (FAZ) area were assessed in 118 healthy Caucasian children aged 4 to 17 years. OCTA scans were obtained using the OCT Topcon Triton^®^ device with 3 × 3 mm and 6 × 6 mm macular cubes. Vascular metrics from the superficial (SCP) and deep capillary plexuses (DCP) were analyzed in relation to demographic, refractive, biometric, and structural OCT parameters. Correlation and multivariate linear regression analyses were performed to evaluate associations. **Results**: Age-stratified reference percentiles for macular VD and FAZ area in SCP and DCP are presented for 118 children. Key associations included: (1) Increased macular thickness correlated with higher VD in the fovea and inner ring (SCP and DCP, all *p* < 0.05); (2) Thicker maculas were associated with smaller FAZ areas (SCP: r = −0.72, DCP: r = −0.58, both *p* < 0.001); (3) Older age was linked to reduced VD in the inner macular ring and smaller FAZ area (SCP and DCP, all *p* < 0.001); and (4) longer axial length correlated with lower central VD (SCP: r = −0.27, DCP: r = −0.37, both *p* < 0.05). No significant sex-based differences were observed. **Conclusions**: This study provides normative OCTA data for macular VD and FAZ area in healthy Caucasian children and identifies key associations with ocular parameters. These findings support improved diagnostic accuracy and clinical decision-making in pediatric retinal evaluation.

## 1. Introduction

Optical coherence tomography angiography (OCTA) is a non-invasive imaging modality that enables quantitative assessment of the macular microvasculature, including the superficial plexus (SCP), deep capillary plexus (DCP), choriocapillaris (CC), and the foveal avascular zone (FAZ) [1,2,3]. Image acquisition of the images is rapid and does not require the administration of contrast agents, thereby eliminating the risk of dye-related adverse reactions [2,4]. These advantages make OCTA particularly suitable for pediatric populations, where it has become an increasingly valuable tool for investigating retinal pathophysiology and supporting the diagnosis and management of various pediatric ocular disorders [1,3].

OCTA has been applied in a wide range of pediatric retinal conditions, encompassing both common and rare entities such as amblyopia, diabetic retinopathy [5,6]; Coats disease [7], retinopathy of prematurity (ROP) [8], Von Hippel Lindau disease, familial exudative vitreoretinopathy (FEVR), incontinentia pigmenti, sickle cell retinopathy, Stargardt Disease, X-linked juvenile retinoschisis, pediatric retinal tumors, and choroidal neovascularization [9,10]. Despite its growing use in pediatric ophthalmology, a major limitation remains the lack of age-specific normative data for macular vascular parameters [3,5]. Existing pediatric reference datasets are predominantly derived from Optovue devices and focus primarily on SCP metrics, with limited and inconsistent reporting for the DCP and across different scan cube sizes (3 × 3 mm and 6 × 6 mm). Moreover, current normative values are largely based on adult populations, which may lead to misclassification when applied to children, as demonstrated by significant discrepancies in percentile-based assessments of retinal parameters [11].

Therefore, the primary objectives of this study were: (1) to establish an age-stratified normative database of macular vessel density (VD) and FAZ area in a cohort of healthy Caucasian children using the Topcon Triton OCTA system, and (2) to evaluate the influence of demographic, refractive, biometric, and structural OCT parameters on macular vascular metrics.

## 2. Methods

### 2.1. Study Design & Ethics

This was a prospective, cross-sectional, single-center study conducted at the Ophthalmology Department of Clínica Universidad de Navarra (Pamplona, Navarra, Spain) between September 2018 and July 2019. Participants included pediatric patients who underwent routine ophthalmologic examinations with normal findings and no history of ocular or systemic disease. The study protocol was approved by the Ethics Committee of Clínica Universidad de Navarra and adhered to the principles of the Declaration of Helsinki and the Good Clinical Practice/International Council for Harmonization (ICH-GCP) guidelines. Written informed consent was obtained from all participants or their legal guardians prior to enrollment. All personal data were anonymized or encrypted to ensure confidentiality and protect patient identity.

### 2.2. Study Population

This study included consecutive pediatric patients aged 4 to 17 years who presented with no ocular pathology at the time of examination and no prior ophthalmic history. Participants were stratified into five age groups. Inclusion and exclusion criteria are detailed in Table 1. All participants underwent a comprehensive ophthalmologic examination of both eyes, including best-corrected visual acuity (Snellen chart), cycloplegic refraction, and anterior and posterior segment evaluation. Axial length (AL) and anterior chamber depth (ACD) were measured using non-contact partial coherence interferometry (IOL Master 500, version 3.01; Carl Zeiss Meditec, Jena, Germany).

### 2.3. Optical Coherence Tomography (OCT)

Structural macular and optic nerve parameters were obtained using swept-source optical coherence tomography (SS-OCT) with the DRI OCT Triton-1 (Topcon, Tokyo, Japan). The following metrics were analyzed: retinal thickness across the nine ETDRS (Early Treatment Diabetic Retinopathy Study) subfields, mean retinal thickness (ETDRS areas A1–A9), and foveal thickness, corresponding to the central 1mm subfield (central retinal thickness, ETDRS area A1). Ganglion cell complex (GCC) thickness was measured in the same ETDRS subfields, including average GCC thickness (areas A1–A9) and central GCC thickness at the foveal center (ETDRS area A1). Additional optic nerve parameters included total retinal nerve fiber layer (RNFL) thickness, RNFL thickness in the temporal, superior, nasal, and inferior quadrants, disc area, optic cup area, neuroretinal rim area, and vertical cup-to-disc ratio (C/D) ratio.

### 2.4. Optical Coherence Tomography Angiography

Macula-centered OCTA scans were acquired in both eyes using the DRI OCT Triton device (Topcon Corporation, Tokyo, Japan), employing both 3 × 3 mm and 6 × 6 mm scan protocols. Structural OCT and OCTA images were processed using the ImageNet 6 software (version 1.20.11109; Topcon Corporation). Quantitative analysis was performed on enface projections of the SCP and DCP. The avascular and choriocapillaris layers were not included in the analysis.

Segmentation of the SCP and DCP was performed automatically using the device’s built-in software. Quantitative data for VD in the SCP were provided directly by the OCTA system, while VD measurements in the DCP were obtained semi-automatically. For DCP segmentation, the inner boundary was set at 15.6 microns below the inner plexiform/inner nuclei layer, and the outer boundary at 70.2 microns, as described elsewhere [12]. Projection artifacts in the DCP were removed using the device’s validated built-in algorithm to enhance image quality. The FAZ area was manually measured using ImageJ software (version 1.53a, Bethesda, National Institutes of Health, Rockville, MD, USA).

Quantitative parameters analysed included FAZ area (mm^2^) and VD (%) in both SCP and DCP, subdivided into foveal and parafoveal regions. VD was defined as the proportion of the area occupied by large and capillary vessels relative to the total area within each region of interest. Enface OCTA images were automatically segmented into standardized ETDRS subfields. VD values were calculated for each subfield, with the fovea corresponding to area A1, inner ring to areas A2–A5 and the outer ring to areas A6–A9.

### 2.5. Statistical Analysis

Statistical analysis was performed using GraphPad Prism software (version 8.0.1, GraphPad Software Inc., San Diego, CA, USA). Descriptive statistics were reported as mean ± standard deviation (SD) or median with interquartile range (IQR), as appropriate. Normality of data distribution was assessed using the Kolmogorov–Smirnov test. Pearson correlation coefficients were calculated for variables with normal distribution, while Spearman’ rank correlation was used for non-normally distributed data. A *p* value of less than 0.05 was considered significant. Sample size was determined based on previous studies addressing normative OCTA databases, with a minimum target of 100 subjects. Multiple linear regression analysis was conducted to identify and quantify independent associations between retinal vascular parameters and structural, clinical and demographic variables, adjusting for potential confounding factors.

## 3. Results

### 3.1. Demographic, Clinical and Structural OCT Characteristics of the Study Cohort

A total of 128 Caucasian children aged 4 to 17 years were recruited for this study. Due to motion artifacts affecting image quality, 10 participants were excluded and a total of 118 participants were included in the final analysis. The cohort consisted of 62 females and 56 males, with both eyes examined. No statistically significant differences were observed between right and left eyes, and one eye per subject was randomly selected for inclusion in the analysis. The main demographic and clinical characteristics of the study population are summarized in Table 2.

### 3.2. OCT Angiography Findings

#### 3.2.1. Macular VD and FAZ Area Metrics

The quantitative measurements of the FAZ area and VD in both the SCP and DCP, obtained using 3 × 3 mm and 6 × 6 mm macular scan protocols, are presented in Table 3. No statistically significant differences were observed between male and female participants across all measured parameters. Similarly, VD values in the inner ring of the DCP showed no significant gender-related variation.

#### 3.2.2. Age-Stratified Vessel Density Percentiles

Table 4 presents the VD percentiles—5th, 25th, 50th, 75th, and 95th—stratified by age group, area of interest (fovea and inner ring), macular cube size (3 × 3 mm and 6 × 6 mm), and vascular plexus (SCP and DCP) in the pediatric cohort. No statistically significant differences were observed between male and female participants across any of the measured parameters. Similarly, inner ring VD values in the DCP did not show significant gender-related variation.

To evaluate VD across age groups, a one-way ANOVA test was applied when data followed a normal (Gaussian) distribution; otherwise, the non-parametric Kruskal–Wallis test was used. No statistically significant differences in VD were observed across age groups in the SCP, either in the foveal area or the inner ring, for both 3 × 3 mm and 6 × 6 mm scan protocols. However, a significant age-related difference was identified in the foveal region of the DCP using the 3 × 3 mm scan protocol (r^2^ = 0.11, F = 3.2, *p* = 0.03). This difference remained statistically significant even after excluding the youngest age group. No other significant age-related differences were found in DCP measurements within the inner ring or foveal area for either scan size.

#### 3.2.3. Correlation Studies and Multivariate Analyses

a. *Associations between age and vascular parameters*

**Figure 1:**
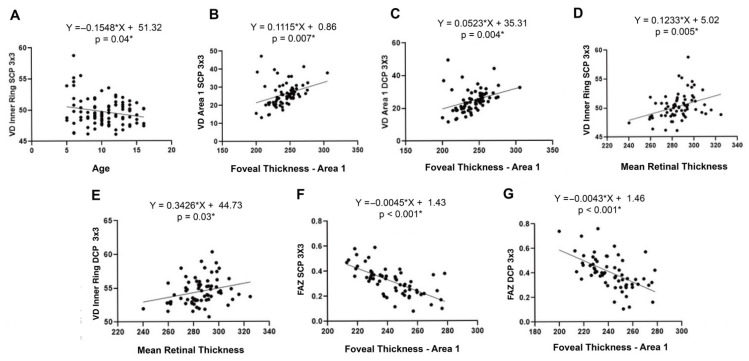
Correlation studies between OCTA metrics and clinical and structural parameters, including age (**A**), foveal thickness (ETDRS area A1) (**B**,**C**,**F**,**G**), and mean retinal thickness (ETDRS areas A1–A9) (**D**,**E**). (VD: vessel density, SCP: superficial capillary plexus, DCP: deep capillary plexus, FAZ: foveal avascular zone area) (* = Significant differences).

Higher VD values were observed in younger participants within the inner macular ring of the SCP (r = −0.208; *p* = 0.04, Figure 1A). In multivariate linear regression analysis, age remained an independent predictor of inner ring VD in the SCP. After adjusting for gender, the association remained statistically significant (β = −0.1553; 95% CI: −0.305 to −0.006; *p* = 0.042), with a model fit of r^2^ = 0.044. Further adjustment for mean retinal thickness improved the model’s predictive power (r^2^ = 0.123), with both age (β = −0.1979; *p* = 0.0241) and retinal thickness (β = 0.0123; *p* = 0.0285) contributing significantly.

Regarding the FAZ area, age was inversely correlated with FAZ area in both SCP and DCP (r = −0.433 and −0.408, respectively; *p* < 0.001). Multivariate analysis adjusting for gender confirmed age as a significant predictor of FAZ area in the SCP (β = −0.0146; *p* = 0.0058; r^2^ = 0.1305). Including foveal thickness further improved model performance (r^2^ = 0.177), with age remaining a significant factor (β = −0.01175; *p* = 0.0287).

b. *Associations between axial length, spherical equivalent, and vascular metrics*

A negative correlation was observed between AL and VD at the fovea, with longer eyes exhibiting lower VD values in both the SCP (r = −0.27, *p* = 0.02) and the DCP (r = −0.37, *p* = 0.001). However, in multivariate regression models adjusting for age, gender, retinal thickness, and spherical equivalent (SE), AL did not remain a statistically significant predictor of VD in either plexus (r^2^ range: 0.04–0.11). No significant correlation was found between AL and FAZ area in either the SCP or DCP.

Regarding SE, a weak positive correlation with FAZ area was noted in the SCP (r = 0.19; *p* = 0.08), while a moderate and statistically significant correlation was observed in the DCP (r = 0.29; *p* = 0.01). In multivariate analysis, SE did not significantly predict FAZ area in the SCP after adjusting for age and gender (r^2^ = 0.1305, *p* = 0.6146). Conversely, SE remained an independent predictor of FAZ area in the DCP (β = 0.01637; *p* = 0.0438), with a modest improvement in model fit (r^2^ = 0.2073). Further adjustment for foveal thickness slightly enhanced the model’s predictive strength (r^2^ = 0.2262), supporting an independent association between more hyperopic refractive error and larger FAZ area in the DCP.

c. *Associations between structural and vascular parameters*

Moderate positive correlations were observed between retinal thickness and VD across multiple macular subfields. In 3 × 3 mm macular scans, the strongest associations were found between foveal thickness (ETDRS area A1) and VD in both the SCP (r = 0.327, *p* = 0.007, Figure 1B) and DCP (r = 0.341, *p* = 0.005, Figure 1C). Mean retinal thickness (ETDRS areas A1–A9) was also positively correlated with VD in the inner macular ring (areas A2–A5) for both SCP (r = 0.343, *p* = 0.004, Figure 1D) and DCP (r = 0.259, *p* = 0.033, Figure 1E). In multivariate linear regression analysis, mean retinal thickness remained a significant independent predictor of inner macular VD in SCP (β = 0.01206, r^2^ = 0.1299, *p* = 0.034) and DCP (β = 0.01075, r^2^ = 0.1316, *p* = 0.03), after adjusting for age, sex, and SE.

Strong inverse correlations were identified between FAZ area and foveal thickness in SCP (r = −0.660, Figure 1F) and DCP (r = −0.566, Figure 1G), both with *p* < 0.001. These associations remained significant in multivariate models adjusted for age and sex, although the inclusion of thickness parameters did not substantially improve predictive power beyond age alone (SCP: r^2^ = 0.1769, *p* = 0.0287; DCP: r^2^ = 0.1666, *p* = 0.009).

Ganglion cell layer (GCL) thickness at the fovea showed positive correlations with VD in both SCP (r = 0.266, *p* = 0.032) and DCP (r = 0.349, *p* = 0.004). Mean GCL thickness was also positively associated with VD in the inner macular ring in SCP (r = 0.345, *p* = 0.004). In multivariate analysis, GCL thickness remained an independent predictor of foveal VD (β = 0.3546, *p* = 0.0037, r^2^ = 0.1401). FAZ area demonstrated strong inverse correlations with GCL thickness in both SCP (r = −0.843, *p* < 0.001) and DCP (r = −0.770, *p* < 0.001).

A statistically significant correlation was found between mean macular RNFL thickness and VD in the inner ring of both SCP (r = 0.267, *p* = 0.01) and DCP (r = 0.205, *p* = 0.048) in 3 × 3 mm scans. Total RNFL thickness showed a weak positive correlation with FAZ area in SCP (r = 0.233, *p* = 0.035). However, RNFL parameters did not retain significance in multivariate models.

## 4. Discussion

Reliable interpretation of OCT and OCTA data in pediatric populations necessitates normative databases specifically tailored for children. In this study, we present reference values for macular VD and FAZ area in Caucasian children, obtained using the Topcon Triton^®^ OCT device (Topcon Corp, Tokyo, Japan). These values are provided in percentiles stratified by age groups, facilitating future comparisons across age-matched cohorts. Additionally, structural retinal data were collected, enabling analysis of key associations between macular vascular parameters and macular thickness, age, sex and AL. Age-specific and device-specific normative datasets enhance reproducibility in research by supporting consistent cross-study comparisons [13], and improve clinical decision-making by offering a healthy reference population for diagnostic evaluation.

Commercially available OCTA devices provide reference ranges derived from adult populations, posing a significant risk of misclassification when applied to pediatric patients. Notably, up to 61% of macular measurements in children may be inaccurately classified when using adult-based normative databases, potentially leading to overdiagnosis or underdiagnosis of retinal pathologies. The importance of age-matched reference data for accurately assessing microvascular retinal metrics—such as VD and FAZ area—even in adult populations, has been highlighted [14]. This challenge is further amplified in pediatric cohorts, where retinal architecture and vasculature are still maturing, rendering adult benchmarks inadequate for detecting deviations from normal development. Equally critical is the need for device-specific normative databases, given the substantial variability in OCTA measurements across imaging platforms [4]. For example, swept-source OCTA typically yields smaller FAZ measurements due to enhanced choroidal penetration and improved segmentation accuracy [5]. Even among devices employing the same imaging technology, measurement discrepancies persist due to differences in scan field size (e.g., 3 × 3 mm vs. 6 × 6 mm), motion artifact correction, and image processing algorithms [6]. Comparative studies between platforms such as the Topcon DRI OCT Triton and Zeiss Cirrus have revealed significant inconsistencies, underscoring the necessity of normative datasets that are both age- and device-specific [13].

Quantification of VD in both the SCP and DCP using OCT angiography (OCTA) is essential for a comprehensive understanding of retinal microvascular architecture and function. Evaluating both plexuses enables accurate assessment of age-related changes, facilitates the diagnosis and monitoring of retinal diseases, and supports the development of robust normative datasets for clinical evaluation. Limiting analysis to a single vascular layer may overlook critical microvascular alterations, particularly in pediatric populations where retinal development is ongoing [15,16,17,18,19]. In our Caucasic cohort, the mean FAZ area was significantly larger in the DCP (0.40 ± 0.13 mm^2^) than in the SCP (0.31 ± 0.12 mm^2^), consistent with previous pediatric studies reporting FAZ values between 0.26 and 0.47 mm^2^ using various OCTA devices and protocols [4,16,20,21,22,23,24,25,26,27,28,29,30,31,32,33,34,35,36] (Table 5). Hsu et al. reported a FAZ of 0.35 ± 0.17 mm^2^ in 89 eyes with a mean age of 8.5 ± 5.3 years, using a Heidelberg OCTA instrument and MATLAB program (MathWorks, Natick, MA, USA), though the evaluated field size was unspecified [26]. In comparison, Yilmaz et al. measured the SCP-FAZ area as 0.28 ± 0.09 mm^2^ and the DCP-FAZ as 0.38 ± 0.09 mm^2^ in 15 eyes from 15 normal children, with a mean age of 8.6 ± 2.2 years, using Nidek’s RS-3000 (Nidek Co., Gamagori, Japan) in a 3 × 3-mm field [37]. İçel et al. recorded the SCP-FAZ as 0.3 ± 0.09 mm^2^ in 146 eyes from children with a mean age of 11.27 ± 3 years, utilizing Nidek RS-3000 AngioScan with a 3 × 3-mm field [28]. Ghassemi et al. reported the mean FAZ area was 0.44 ± 0.78 mm^2^, measured in a single layer from a 3 × 3-mm enface image using automated software [30]. No significant gender differences were observed in our series, in line with previous reports [37]. Variability across studies may reflect differences in sample size, imaging devices, vascular layer analyzed, and ethnicity, which is very relevant as retinal blood vessels vary between ethnic groups. Notably, FAZ values in healthy children are generally consistent with those reported in adults, where larger areas are typically observed in the DCP [19,38].

In our cohort, gender did not significantly influence VD or FAZ area across macular regions, despite girls exhibiting greater macular thickness than boys. This finding contrasts with previous studies that reported larger FAZ in DCP in boys [39]. The absence of gender-related differences in our sample may be explained by the young age of participants, prior to the onset of puberty, when hormonal effects on retinal vasculature are likely minimal [40]. Our findings indicate a significant inverse correlation between age and vessel density (VD) in the inner macular ring, both in the superficial capillary plexus (SCP) (r = −0.208; *p* = 0.04) and deep capillary plexus (DCP) (r = −0.408; *p* < 0.001). This age-related decline in VD is consistent with previous studies and may reflect physiological vascular remodeling, reduced metabolic demand, and capillary dropout associated with retinal maturation [37,41].

Similarly, a moderate negative correlation was observed between age and foveal avascular zone (FAZ) area in both the SCP (r = −0.433; *p* < 0.001) and DCP (r = −0.408; *p* < 0.001). These findings support the notion that FAZ size decreases during childhood as retinal vascularization progresses and oxygen demand increases. Campbell et al. previously demonstrated age-dependent reductions in FAZ size in healthy children, highlighting the role of retinal vascular development in neurophysiological maturation [3]. Importantly, the association remained significant after adjusting for gender and foveal thickness, reinforcing age as a primary determinant of FAZ morphology.

Our study identified a significant inverse correlation between AL and VD in the central macular region, both in the SCP (r = −0.27; *p* = 0.02) and DCP (r = −0.37; *p* < 0.001). These findings are consistent with previous reports linking increased AL and myopia to microvascular attenuation, likely due to mechanical retinal stretching and subsequent capillary rarefaction [42]. Al-Sheikh et al. and Zheng et al. similarly reported reduced macular VD in myopic eyes, particularly within the SCP and DCP [5,43]. In contrast, no significant correlation was observed between AL and FAZ area in either plexus, aligning with prior studies suggesting that FAZ size remains relatively stable despite biometric variations [20]. This stability implies that FAZ morphology may be predominantly influenced by genetic or early developmental factors rather than axial elongation. Multivariate analysis revealed that AL and SE had limited predictive value for VD and FAZ area. While biometric parameters may exert some influence on retinal vascular features, their impact appears secondary to structural retinal characteristics and age. These findings support the notion that retinal perfusion and vascular development are more strongly governed by anatomical and maturational factors than by refractive status alone.

Our study demonstrated a significant positive correlation between foveal retinal thickness and VD in both the SCP and DCP (r = 0.343, *p* < 0.004; r = 0.259, *p* < 0.033), independent of age and sex. These findings suggest that retinal thickness is a robust predictor of VD in pediatric populations, consistent with prior studies in both children and adults. Given that the GCC comprises the innermost retinal layers, including the nerve fiber layer, ganglion cell layer, and inner plexiform layer, its thickness significantly influences overall retinal thickness [44]. This relationship has been emphasized in glaucoma research, where GCC measurements are used diagnostically [45]. In our cohort, a thicker GCC was associated with higher VD after adjusting for age and sex. Additionally, higher mean retinal nerve fiber layer (RNFL) thickness correlated with increased VD in the inner macular ring of both SCP (r = 0.267, *p* = 0.01) and DCP (r = 0.205, *p* = 0.048). We also observed strong inverse correlations between foveal thickness and FAZ area (SCP: r = −0.723, *p* < 0.001; DCP: r = −0.581, *p* < 0.001), as well as between GCC thickness at the fovea and FAZ area (SCP: r = −0.843, *p* < 0.001; DCP: r = −0.770, *p* < 0.001). Similar patterns were found when analyzing GCC thickness at the thinnest point of the fovea (SCP: r = −0.682, *p* < 0.001; DCP: r = −0.536, *p* < 0.001). These associations likely reflect normal foveal development and its structural remodeling, underscoring the interplay between neuroretinal architecture and macular vascularization [20]. Our findings are in line with other previous studies in adults, such as those by Samara et al. and Morales et al., which reported a significant inverse relationship between FAZ area and central macular thickness (*p* < 0.001) [20,46].

This study has several limitations. First, as in other pediatric OCTA research, scan repeatability and reproducibility are challenged by variability in fixation and cooperation, potentially affecting data consistency. Although acceptable, repeatability in children remains lower than in adults [1,2]. Moreover, we reported a 7.8% of scanned eyes excluded from analysis due to motion artifacts affecting image quality (10/128), which are common in children. Second, macular measurements tend to be less reliable than peripapillary ones, likely due to anatomical complexity and longer acquisition times [3]. Third, imaging was performed at varying times of day, without controlling for diurnal variation in vascular parameters, which may have introduced intra-individual variability [4]. Additionally, manual segmentation of the FAZ, along with the absence of magnification correction based on axial length or refraction, may have affected measurement precision [3,5,6,47]. Fourth, the image quality threshold selected of >45 is slightly lower than other studies that use a lower limit of >50 or>60. While this minor difference may not have affected the results of the study, it could limit the generalizability of the results and comparisons with other study cohorts. Fifth, the cross-sectional design and the single ethnicity (Caucasians) also limit the generalizability of the results. Future longitudinal study designs including larger sample sizes from multi-ethnic populations are required to overcome these limitations. Finally, the lack of perinatal and gestational history data limits our ability to account for early-life influences on foveal vascular development. Despite these limitations, the integration of structural and vascular metrics in our study provides a comprehensive assessment of macular status in children.

Beyond the scope of this study, several limitations regarding the use of OCTA in pediatric populations warrant consideration. Image acquisition in very young children (under 4 years of age) remains particularly challenging, highlighting the need for the development of handheld, portable OCT devices capable of facilitating supine examinations [48]. Future research directions may focus on the potential of pediatric OCTA metrics to detect early microvascular alterations associated with systemic conditions, such as diabetes mellitus or congenital vascular malformations.

This study provides normative OCTA metrics for retinal VD and FAZ area in a cohort of Caucasian children aged 4–17 years, using the Topcon DRI OCT Triton platform. Percentile-based reference values were established across age groups for both the SCP and DCP to establish a range of values that may serve as thresholds for raising concerns in the presence of outliers. VD was higher in the SCP at the fovea and in the DCP within the parafoveal region, while FAZ areas were consistently larger in the DCP. Increased macular and ganglion cell complex (GCC) thickness correlated positively with VD and inversely with FAZ area. Age and AL demonstrated significant negative associations with VD. These findings underscore the relevance of integrating structural and vascular parameters in pediatric OCTA analysis. We propose the systematic integration of these metrics in future studies involving larger and ethnically diverse cohorts, in order to enhance methodological rigor and improve the interpretability and generalizability of OCTA-derived data in both clinical and research settings. This strategy is intended to facilitate the incorporation of OCT and OCTA into routine pediatric ophthalmologic assessments in the near future.

## Figures and Tables

**Table 1:** Inclusion and exclusion criteria.

Inclusion Criteria	Exclusion Criteria
Visual Acuity (Decimal VA) - 5–6 years: ≥0.7 - 7–8 years: ≥0.8 - 9–17 years: ≥0.9	- Amblyopia - Any ocular disease - Any systemic disease - Poor quality scans (defined as Topcon Quality index score < 45, scale 0–100) - Artifacts (motion, blink)
Spherical equivalent (SE) - −6.00 to +5.00 diopters (D)	
Astigmatism - <3.00 D	

**Table 2:** Overview of the main demographic and structural OCT parameters of the study population.

	Study Sample (n = 118)	Boys (n = 56)	Girls (n = 62)	*p*
Age, years				
Mean ± SD	10 ± 3	10 ± 2.7	10 ± 3.3	*p* = 0.72
Median (IQR)	10 (5 to 17)			
Sex, n (%)				
Girls	62 (52.5%)	56 (47.5%)	62 (52.5%)	*p* = 0.7
Boys	56 (47.5%)			
BCVA, logMAR				
Mean ± SD	1 ± 0.057	1.0 ± 0.06	1.0 ± 0.056	*p* = 0.52
Median (IQR)	1.00 (0.70 to 1.00)			
AL, mm				
Mean ± SD	23 ± 1.3	24 ± 1.1	23.19 ± 1.33	*p* = 0.03 *
Median (IQR)	23 (20–26 mm)			
K1, D				
Mean ± SD	43 ± 1.6	42.50 ± 1.62	43.25 ± 1.44	*p* = 0.01 *
Median (IQR)	43 (38 to 48)			
K2, D				
Mean ± SD	44 ± 1.7	43.63 ± 1.64	44.39 ± 1.59	*p* = 0.01 *
Median (IQR)	44 (40 to 48)			
ACD, mm				
Mean ± SD	3.5 ± 0.31	3.56 ± 0.28	3.53 ± 0.32	*p* = 0.57
Median (IQR)	3.5 (2.8 to 4.3)			
Spherical Equivalent, D				
Mean ± SD	+0.036 ± 2.3	0.01 ± 2.4	0.06 ± 2.2	*p* = 0.89
Median (IQR)	+0.50 (−6.00 to +4.80)			
Average macular thickness				
Mean ± SD	259 ± 64	238 ± 57	275 ± 64	
Median (IQR)	263 (134 to 402)	255 (134 to 336)	263 (170 to 402)	*p* = 0.02 *
Central macular thickness				
Mean ± SD	279 ± 73	251 ± 63	300 ± 74	*p* = 0.001 *
Median (IQR)	280 (141 to 469)	264 (141 to 355)	301 (149 to 469)	
GCL average thickness				
Mean ± SD	74 ± 5.2	74 ± 4.9	74 ± 5.5	*p* = 0.77
Median (IQR)	74(58 to 85)	75 (63 to 84)	77 (58 to 85)	
GCL central thickness				
Mean ± SD	13 ± 7.4	14 ± 7.3	12 ± 7.4	*p* = 0.33
Median (IQR)	12(7.3 to 35)	15 (8 to 28)	12 (6 to 35)	
RNFL macula layer				
Mean ± SD	110 ± 9.9	111 ± 9.9	110 ± 10	*p* = 0.68
Median (IQR)	111(88 to 139)	111(88 to 139)	110 (88 to 132)	

ACD: Anterior chamber depth; AL: Axial length; BCVA: Best corrected visual acuity; D: Diopters; GCL: Ganglion cell complex; IQR: Interquartile range; SD: Standard deviation. (* = Significant differences).

**Table 3:** VD figures in the SCP vs. DCP (mean and SD) for the area of interest in the macular region (fovea or inner ring). Sizes of macular cubes: 3 × 3 and 6 × 6 mm. FAZ area metrics in both plexi from Macular cube 3 × 3 mm. All variables are presented as mean ± SD.

	3 × 3 mm						6 × 6 mm			
	SCP			DCP			SCP		DCP	
	Fovea	Inner Ring	FAZ	Fovea	Inner Ring	FAZ	Fovea	Inner Ring	Fovea	Inner Ring
Overall	25.93 ± 5.9	49.71 ± 2.1	0.31 ± 0.12	24.78 ± 6.2	53.91 ± 2	0.4 ± 0.13	25.04 ± 5.8	49.09 ± 2.7	23.91 ± 6	48.01 ± 4
Boys	26.40 ± 5.1	49.77 ± 1.9	0.31 ± 0.1	24.99 ± 5.5	53.68 ± 2	0.4 ± 0.1	25.71 ± 5.6	49.30 ± 3.3	24.49 ± 5.9	47.91 ± 4.9
Girls	25.48 ± 6.5	49.65 ± 2.4	0.29 ± 0.11	24.58 ± 7	54.13 ± 2	0.39 ± 0.1	24.42 ± 5.94	48.9 ± 2.1	23.38 ± 6.1	48.10 ± 3
*p* (Boys vs. Girls)	*p* = 0.45	*p* = 0.79	*p* = 0.43	*p* = 0.75	*p* = 0.28	*p* = 0.69	*p* = 0.21	*p* = 0.56	*p* = 0.22	*p* = 0.96

**Table 4:** Percentiles of VD for a paediatric cohort.

		SCP 3 × 3						DCP 3 × 3					
Age	Area	x ± SD	P5	P25	P50	P75	P95	x ± SD	P5	P25	P50	P75	P95
<7 (n = 25)	Fovea	29 ± 9	14.66	21.46	28.07	37.75	47.13	28 ± 9.8	13.12	18.78	25.51	34.83	49.54
	Inner Ring	51 ± 3.4	46.28	48.40	50.37	53.96	58.75	55 ± 3	50.41	53.15	54.48	57.92	60.36
8–9 (n= 25)	Fovea	24 ± 5.9	13.31	20.03	23.73	28.23	37.44	21.4 ± 5.4	11.83	17.69	21.04	24.84	33.11
	Inner Ring	49.3 ± 1.5	46.20	48.67	49.10	50.49	51.95	53.7 ± 1.5	50.20	52.81	53.59	54.82	56.66
10–11 (n = 27)	Fovea	25.3 ± 4.5	17.53	20.97	25.80	28.50	35.18	24.2 ± 4.5	16.09	20	24.70	28.44	32.21
	Inner Ring	49.3 ± 1.6	47.46	48.02	49.06	50.11	53.48	53.5 ± 1.8	50.54	52.65	53.08	53.88	58.40
12–13 (n = 22)	Fovea	25.5 ± 3.1	18.92	24.09	25.44	29.18	29.55	24.8 ± 3.4	17.24	22.80	25.43	27.90	29.12
	Inner Ring	49.7 ± 1.9	46.09	48.57	50.09	51.20	52.38	54 ± 1.6	51.36	52.89	54.00	55.28	56.22
>14 (n = 19)	Fovea	26.1 ± 4.4	14.84	23.62	26.07	29.43	32.36	26.1 ± 5	13.68	22.65	27.10	29.92	33.85
	Inner Ring	49.3 ± 1.3	47.35	47.82	49.52	50.21	51.45	43.4 ± 1.6	50.45	51.88	53.62	54.61	55.85
		SCP 6 × 6						DCP 6 × 6					
Age	Area	x ± SD	P5	P25	P50	P75	P95	x ± SD	P5	P25	P50	P75	P95
<7 (n = 25)	Fovea	29 ± 9.1	17.45	22.29	26.31	33.30	49.73	28.03 ± 9.9	16.21	20.59	24.48	33.77	50.59
	Inner Ring	50 ± 4.7	45.35	47.55	49.90	52.49	65.19	48 ± 6.5	27.18	46.91	48.13	49.95	57.47
8–9 (n = 25)	Fovea	22.5 ± 4.8	12.99	19.29	22.74	27.03	28.83	21.3 ± 4.6	12.47	17.89	21.31	24.79	29.19
	Inner Ring	48.9 ± 1.6	45.79	47.99	48.50	50.08	52.75	48.2 ± 3.47	44.64	46.02	46.57	48.98	57.25
10–11 (n = 27)	Fovea	24.7 ± 4.41	18.44	20.33	24.47	27.76	34.55	23.2 ± 4	16.61	16.93	23.20	26.21	29.78
	Inner Ring	48.6 ± 2.2	44.88	47.28	48.50	50.12	53	47.7 ± 3.18	43.59	46.02	46.70	48.66	56.18
12–13 (n = 22)	Fovea	24.2 ± 2.92	18.60	22.19	23.97	25.22	29.92	23.1 ± 3.54	16.52	20.50	23.17	25.51	29.90
	Inner Ring	48.5 ± 1.40	46.53	47.25	48.20	49.49	51.27	48.1 ± 3.07	44.96	45.83	46.95	48.77	54.74
>14 (n = 19)	Fovea	25 ± 3.5	16.58	23.09	24.81	27.13	31.29	24.3 ± 3.7	15.45	21.93	23.79	27.33	30.72
	Inner Ring	49 ± 2.2	43.40	47.44	49.31	50.22	53.44	48.2 ± 2.8	43.51	46.82	47.69	49.44	57.17

**Table 5:** Summary of publications about OCTA metrics in pediatric populations. º means “degrees”.

Author	Year	Device	Software (Version)	Scan (mm)	Sample Size (n)	Age (years)	Ethnicity	SCP (mean ± SD)	DCP (mean ± SD)	FAZ mm^2^ (SCP or DCP)
Zhang et al. [16]	2017	Optove RTVue Avanti	AngioVue (v2016.1.0.26)	3 × 3	75	8–16	Asian (Chinese)	54.29 ± 2.25 VD%	60.19 ± 1.76 VD%	SCP: 0.29 ± 0.109
Hsu et al. [26]	2019	Spectralis	MATLAB *	10º × 10º	89	9 weeks–17	Caucasian, African, Hispanic, Asian, other	35 ± 2.5 VAD% 28.2 ± 3.3 VLD%	39.4 ± 1.5 VAD% 33.1 ± 2.1 VLD%	DCP: 0.35 ± 0.17
Borrelli et al. [21]	2019	Optove RTVue Avanti	AngioVue (v2016.1.0.26)	3 × 3	77	5–17	Caucasian, African, Hispanic, Asian	0.33 ± 0.24 PD% 0.12 ± 0.01 VD%	0.35 ± 0.13 PD% 0.13 ± 0.01 VD%	SCP: 0.261 ± 0.149
Zhang et al. [27]	2020	Optove RTVue Avanti	AngioVue (v2018.0.0.18)	6 × 6	71	4.5–20.5	Asian (Chinese)	20.10 ± 7.13 VD%	36.19 ± 7.68 VD%	SCP: 0.28 ± 0.10
Içel E et al. [28]	2020	Nidek RS-3000	Angioscan *	3 × 3	146	6–16	Not specified	43.88 ± 3.4 VD%	39.6 ± 3.55 VD%	SCP: 0.3 ± 0.09
Li S et al. [29]	2020	Zeiss Cirrus 5000	AngioPlex *	3 × 3	333	4–16	Asian (Chinese)	4–6.9 years: 20.81 ± 1.56 7–9.9 years: 21.57 ± 1.05 10–12.9 years: 21.61 ± 1.36 13–15.9 years: 22.01 ± 0.89		SCP 4–6.9 years: 0.22 ± 0.084 7–9.9 years: 0.24 ± 0.085 10–12.9 years: 0.24 ± 0.093 13–15.9 years: 0.25 ± 0.089
Ghassemi et al. [30]	2021	Optovue RTVue XR Avanti	AngioVue (v2016.1.0.23)	6 × 6	54 patients (108 eyes)	3–18	Not specified	Total: 50.66 (21.04–56.74) VD% <7 years: 51.83 (21.04–56.74) VD% 7–10 years: 51.76 (44.09–55.29) VD% 11–14 years:51.17 (46.58–55.68) VD% >14 years: 49.53 (35.81–55.01) VD%	Total: 51.15 (16.36–63.32) VD% <7 years: 50.23 (16.36–63.32) VD% 7–10 years: 51.41 (43.53–61.12) VD% 11–14 years: 52.93 (42.62–61.51) VD% >14 years: 49.14 (28.51–58.65) VD%	SCP Total:0.28 (0.04–4.20) <7 years: 0.27 (0.05–1.10) 7–10 years: 0.28 (0.10–5.05) 11–14 years: 0.29 (0.09–0.40) >14 years: 0.28 (0.04–4.20)
Xiang et al. [31]	2021	Optove RTVue Avanti	AngioVue (v2017.1.0.155)	6 × 6	242	4–6	Asian (Chinese)	48.10 ± 2.92	48.74 ± 6.51	SCP 0.3 ± 0.13
Kurumoğlu Incekalan et al. [32]	2021	Optove RTVue Avanti	AngioVue (v2017.1.0.151)	3 × 3	185	7–18	Not Specified	7–9 years: 18.22 ± 6.1 VD% 10–12 years: 18.71 ± 4.77 VD% 13–15 years: 18.69 ± 5.77 VD% 16–18 years: 18.34 ± 5.27 VD%	7–9 years: 35.16 ± 7.58 VD% 10–12 years: 35.48 ± 6.88 VD% 13–15 years: 35.43 ± 7.14 VD% 16–18 years: 35.54 ± 5.96 VD%	SCP 7–9 years: 0.28 ± 0.09 10–12 years: 0.27 ± 0.11 13–15 years: 0.27 ± 0.11 16–18 years: 0.26 ± 0.08
Bajtl et al. [33]	2021	Spectralis OCTA	AngioTool (v0.6a,02.18.14)	10º × 10º	62	4–5	Caucasian	Boys: 65.12 Girls: 64.84	Boys: 68.02 Girls: 66.81	SCP Boys: 0.55/Girls: 0.52 DCP Boys: 0.52/Girls: 0.53
Chen R et al. [34]	2022	Optove RTVue Avanti	AngioVue (v2018.0.0.14)	6 × 6	370	7–18	Not Specified	7–9 years: 18.22 ± 6.1 VD% 10–12 years: 18.71 ± 4.77 VD% 13–15 years: 18.69 ± 5.77 VD% 16–18 years: 18.34 ± 5.27 VD%	7–9 years: 35.16 ± 7.58 VD% 10–12 years: 35.48 ± 6.88 VD% 13–15 years: 35.43 ± 7.14 VD% 16–18 years: 35.54 ± 5.96 VD%	SCP 7–9 years: 0.28 ± 0.09 10–12 years: 0.27 ± 0.11 13–15 years: 0.27 ± 0.11 16–18 years: 0.26 ± 0.08
Plaitano et al. [35]	2022	DRI SS-OCT-A Triton plus	IMAGENET 6	4.5 × 4.5	206	4–16	Caucasian	Fovea 17.1 ± 4.26 PD%	Fovea 13.55 ± 5.23 PD%	SCP:234 ± 106.39 DCP:298.32 ± 112.37
Diao et al. [36]	2023	Optove RTVue XR-Avanti	AngioVue *	6 × 6	90	6–15	Asian (Chinese)	Whole retina: 49.934 ± 2.362 VD% Fovea: 22.668 ± 6.728 VD% Parafovea: 52.536 ± 3.083 VD% Perifovea: 50.570 ± 2.473 VD%	Whole retina 55.015 ± 4.764VD% Fovea: 35.155 ± 7.636 VD% Parafovea: 54.676 ± 5.676 VD% Perifovea: 56.373 ± 5.353 VD%	SCP 0.261 ± 0.104
*THIS STUDY* *Guirao-Navarro et al.*	*2025*	*DRI OCT Triton*	*IMAGENET 6*	*3 × 3* *6 × 6*	*118*	*4–17*	*Caucasian*	*3 × 3* *A1: 25.93 ± 5.9* *AI: 39.71 ± 2.1* *6 × 6* *A1:25.04 ± 5.8* *AI: 49.09 ± 2.7*	*3 × 3* *A1: 24.78 ± 6.2* *AI: 53.91 ± 2* *6 × 6* *A1:23.91 ± 6* *AI: 48.01 ± 4*	***3 × 3*** ***SCP: 0.31 ± 0.12*** ***DCP: 0.40 ± 0.13***

* = Software version not provided.

## Data Availability

Data will be made available upon request from the corresponding author. The data are not publicly available due to privacy concerns.

## Abbreviations

The following abbreviations are used in this manuscript (in alphabetical order):

ACD	Anterior chamber depth
AL	Axial length
CC	Choriocapillaris
DCP	Deep capillary plexus
ETDRS	Early treatment diabetic retinopathy study
FAZ	Foveal avascular zone
FEVR	Familial exudative vitreoretinopathy
GCC	Ganglion cell complex
IQR	Interquartile range
OCT	Optical Coherence Tomography
OCTA	Optical Coherence Tomography Angiography
RNFL	Retinal nerve fiber layer
ROP	Retinopathy of prematurity
SCP	Superficial capillary plexus
SD	Standard deviation
SE	Spherical equivalent
VD	Vessel density
